# The GLV6/RGF8/CLEL2 peptide regulates early pericycle divisions during lateral root initiation

**DOI:** 10.1093/jxb/erv329

**Published:** 2015-07-10

**Authors:** Ana Fernandez, Andrzej Drozdzecki, Kurt Hoogewijs, Valya Vassileva, Annemieke Madder, Tom Beeckman, Pierre Hilson

**Affiliations:** ^1^Department of Plant Systems Biology, VIB, B-9052 Ghent, Belgium.; ^2^Department of Plant Biotechnology and Bioinformatics, Ghent University, B-9052 Ghent, Belgium.; ^3^Department of Organic Chemistry, Ghent University, 9000 Ghent, Belgium.; ^4^Institute of Plant Physiology and Genetics, Bulgarian Academy of Sciences, 1113 Sofia, Bulgaria.; ^5^INRA, UMR1318, Institut Jean-Pierre Bourgin, RD10, F-78000 Versailles, France.; ^6^AgroParisTech, Institut Jean-Pierre Bourgin, RD10, F-78000 Versailles, France.

**Keywords:** Asymmetric division, *Arabidopsis thaliana*, CLE-like, GOLVEN, lateral root development, primordium initiation, root growth factors, secreted peptides.

## Abstract

Small peptides of the *Arabidopsis* GLV/RGF/CLEL family are involved in different developmental programmes, including meristem maintenance and gravitropic responses. In addition, our previous report suggested that they also participate in the formation of lateral roots. Specifically, *GLV6* is transcribed during the first stages of primordium development and *GLV6* overexpression results in a strong reduction of emerged lateral roots. To investigate the cause of this phenotype we analysed primordium development in gain-of-function (*gof*) mutants and found that *GLV6* induces supernumerary pericycle divisions, hindering the formation of a dome-shaped primordium, a prerequisite for successful emergence. The *GLV6* phenotype could be reproduced by ectopic expression of the gene only in xylem-pole pericycle cells. Furthermore, GLV6 seems to function at the very beginning of lateral root initiation because GLV6 excess—either gene overexpression or peptide treatment—disrupts the first asymmetric cell divisions required for proper primordium formation. Our results suggest that GLV6 acts during lateral root initiation controlling the patterning of the first pericycle divisions.

## Introduction

Unlike animals, plant organs are initiated in the meristems during post-embryonic development. Meristematic cells proliferate in an undifferentiated state but also produce differentiating cell lineages that eventually give rise to new lateral organs. This process is highly regulated and relies on a complex network of genetic and hormonal cues. In the root, specific pericycle cells at the xylem pole retain the capacity to resume meristematic activity after leaving the root apical meristem (RAM) (Beeckman *et al.*, 2001). As the main root keeps growing, some xylem pole pericycle (XPP) cells undergo a sequence of events resulting in the formation of a novel lateral root primordium (LRP), followed by the emergence of the lateral root (LR) out of the main root tissues.

The earliest event, known to date, leading to LR formation is a regular auxin pulse occurring in the upper portion of the meristematic zone (the basal meristem) that ‘primes’ pericycle cells (De Smet *et al.*, 2007) making them competent to produce LRs. Recently a gene transcription oscillatory mechanism was found to operate in a region of the basal meristem and the elongation zone, termed the oscillation zone, to eventually establish the so-called branching points. This oscillating transcriptional mechanism is thought to define periodic LR formation (Moreno-Risueno *et al.*, 2010). Later on, some primed XPP cells are specified as lateral root founder cells (LRFCs). These cells are usually arranged as pairs of adjacent cells present in two or three contiguous cell files and undergo a series of divisions in the maturation zone to generate a LRP (Casimiro *et al.*, 2001).

The lateral root initiation process starts with the first division of LRFCs. Prior to this initial division, the LRFC nuclei move towards each other, then both cells divide asymmetrically in the anticlinal direction yielding two small central cells and two large peripheral cells (Casimiro *et al.*, 2001; De Smet *et al.*, 2008; De Rybel *et al.*, 2010). Auxin transport and transcriptional response components are required for this process (Benkova *et al.*, 2003; Fukaki *et al.*, 2005; Ditengou *et al.*, 2008; Dubrovsky *et al.*, 2008). A second round of anticlinal divisions yields the first primordium layer. The initial anticlinal divisions generate a recognizable hallmark that is referred to as a stage I primordium (Malamy and Benfey, 1997). The division plane then changes orientation and periclinal divisions result in primordia with an additional cell layer (stage II). Subsequent anticlinal and periclinal divisions generate a dome-shaped structure that eventually emerges from the primary root. Despite extensive studies, the LR formation process (reviewed in Van Norman *et al.*, 2013 and Atkinson *et al.*, 2014) remains poorly understood and the signalling network that controls patterning at early stages of primordium initiation is largely uncharacterized.

Over the last two decades it became clear that small secreted peptides carry cell-to-cell signals in a wide range of plant developmental processes, including root and shoot meristem homeostasis, defense, abscission, vascular and embryonic tissue differentiation, and stomata development (reviewed in Murphy *et al.*, 2012 and Grienenberger and Fletcher, 2015). Several peptide families have recently been implicated specifically in LR development (reviewed in Delay *et al.*, 2013). For example, members of the INFLORESCENCE DEFICIENT IN ABSCISSION (IDA) family, binding to the HAESA and HAESA-LIKE2 receptors, promote the separation of the outer cell layers in the main root to open the path to the emerging LR (Kumpf *et al.*, 2013).

Signalling peptides belonging to the GOLVEN/root growth factor/CLE-like (GLV/RGF/CLEL) family are known regulators of RAM maintenance in *Arabidopsis thaliana*. The GLV11/RGF1, GLV5/RGF2 and GLV7/RGF3 peptides participate in RAM homeostasis via the positive regulation of the PLETHORA (PLT) 1 and 2 transcription factors at both the transcriptional and post-transcriptional levels (Matsuzaki *et al.*, 2010). In addition, GLV/RGF/CLEL peptides are involved in root gravitropic responses since they modulate the turnover of the auxin efflux carrier PIN2 and thus control auxin fluxes in the root tip (Whitford *et al.*, 2012). We showed in a recent report that the overexpression of several *GLV* genes also results in a strong decrease in emerged lateral root (ELR) density as observed independently by another research group for *GLV1/CLEL6* and *GLV10/CLEL8* (Meng *et al.*, 2012). However, the precise function of GLV peptides during LR formation has yet to be defined. Furthermore, the phenotypes resulting from constitutive gene overexpression must be interpreted with caution. For example, *GLV1* is only transcribed in above-ground tissues (Whitford *et al.*, 2012) even though its constitutive overexpression alters LR development (Meng *et al.*, 2012; Fernandez *et al.*, 2013*a*). Because the bioactive mature peptides cleaved off the precursor proteins belonging to the same family are usually highly similar, they may be recognized by receptors to which they are normally not exposed, thereby generating ectopic effects.

Our systematic transcriptional analysis of all eleven *GLV* genes revealed that *GLV6* is the only member of the family already expressed in stage I LRPs and its overexpression resulted in the strongest LR phenotype (Fernandez *et al.*, 2013*a*). The early *GLV6* transcriptional pattern and gain-of-function (*gof*) phenotype suggest a function during the initial steps of primordium formation. Therefore, in this report, we investigate the role of GLV6 during LR initiation.

## Materials and methods

### Plant material and growth conditions

*Arabidopsis thaliana* seedlings were grown on half-strength Murashige and Skoog medium (MS; Duchefa Biochemie B.V.) supplemented with 1% (w/v) sucrose and 1% (w/v) agarose, at pH 5.8 and germinated in a growth chamber at 22°C under continuous light conditions (100 μmol m^-2^ s^-1^). The lines *GATA23pro:NLS-GFP-GUS* (De Rybel *et al.*, 2010), *35Spro:GLV6* (Fernandez *et al.*, 2013*a*), *PIN1pro:PIN1-GFP* (Benkova *et al.*, 2003) and Wave 131Y, containing a plasma membrane-localized YFP (Geldner *et al.*, 2009) have been previously described. *GLV6pro::NLS-GFP-GUS* plants including the *GLV6* promoter driving nuclear *GFP/GUS* expression (Fernandez *et al.*, 2013*a*) were crossed to Wave 131Y and F1 seedlings were used to detect *GLV6* transcription in the developing LRP. Enhancer trap lines are part of the Haseloff collection and were donated or ordered from NASC.

For *GLV6* overexpression in different root cell layers, an Upstream Activating Sequence (UAS) was fused to the *GLV6* open reading frame (ORF) via Gateway (Geldner *et al.*, 2009) (Invitrogen) by combining the pEN-L1-*GLV6*-L2, PEN-L4-*UAS*-R1 and pB7m24GW vectors (Geldner *et al.*, 2009). The resulting *UASpro:GLV6* construct was transformed in enhancer trap lines specific for different root cell layers and T3 single locus homozygous plants were obtained. An empty vector was used as control. All constructs were also transformed in the C24 wild-type background where no transactivation occurs.

Truncated *GLV6* ORFs were amplified and cloned into pDONR221 by Gateway BP reaction (Invitrogen). The *GLV6∆VR* amplicon flanked by Gateway *attL1 and attL2* sites was generated using overlapping PCR as previously described (Atanassov *et al.*, 2009) (see Supplementary Table S1 for primer sequences). Overexpression constructs were generated with the FAST vector (Shimada *et al.*, 2010) which contains a GFP seed marker to select transformants. T1 GFP positive seeds were selected and grown to quantify LR density.

*GLV6* artificial microRNAs *(amiRglv6*) were designed using the Web MicroRNA Designer (WMD1 for *amiRglv6_V1*: TACTACGTTACTACAACCGAT and WMD3 for *amiRglv6_V2*: TAAACTACGTTACTACAACCG and *V3*: TCTAAACGTA CGATGATCCAT) (http://wmd3.weigelworld.org/cgi-bin/webapp.cgi). The *amiRglv6* sequences were cloned into pDONR221 as described in Whitford *et al.* (2012) and each resulting entry clone was assembled with the *GLV6* promoter (pEN-L4-*GLV6pro*-R1) (Fernandez *et al.*, 2013*a*) by LR reaction into the pB7m24GW-FAST (Shimada *et al.*, 2010) binary vector. Single locus homozygous lines where the gene was silenced (Supplementary Table S2) were obtained for each *amiRglv6* construct. Two lines were used for further analysis of root length showing consistent results. Lateral root density was quantified in one line per *amiRglv6* construct.

### Root bending experiments

For induction of lateral root initiation *GATA23pro:NLS-GFP-GUS* seedlings were germinated on MS medium. Four days-after-germination (dag) seedlings were placed in a chambered coverglass (Thermo Scientific Nunc) and the root tip was mechanically bent with flat tweezers between the elongation and the maturation zone. A piece of gelled MS medium, supplemented or not with a peptide, was then immediately placed on top. After bending, roots were imaged in a Zeiss LSM5 confocal microscope every 5min for 14−16h. On average, 30min elapsed from the preparation of the sample up to the first image, therefore t_0_ should be considered as 30min after bending.

### Peptide treatments

GLV6 peptides carrying different post-translational modifications were synthesized as previously described (Whitford *et al.*, 2012). The following peptide sequences were assayed: GLV6p SO_3_, DY(SO_3_)RTFRRRRPVHN; rGLV6p SO_3_, NRRY(SO_3_)RHRFTVDPR and GLV6p Hyp SO_3_, DY(SO_3_)RTFRRRRHypVHN [Y(SO_3_), sulfonated tyrosine; Hyp, hydroxyproline]. Peptide treatments at the indicated concentrations were carried out in liquid to measure ELR density or on solid media for the analysis of the first asymmetric divisions in *PIN1pro:PIN1-GFP* roots.

### Morphological analysis

ELRs were quantified in 8−12 dag seedlings and normalized by the root length (measured with the ImageJ software) to obtain the ELR density. Quantification of total lateral roots (non-emerged and emerged) was carried out in 8−9 dag seedlings after root clearing (Malamy and Benfey, 1997). For transactivation lines, initiation sites were considered to have restricted anticlinal divisions when the primordium border could be detected within the microscope field at 40× magnification.

### Microscopic analysis

Confocal images were taken on a Zeiss LSM5 or 710 confocal microscope. GFP/YFP was detected with a 488nm filter for excitation and 520nm for detection. DIC pictures were obtained with an Olympus microscope (DIC-BX51) equipped with a CAMEDIA C-3040 zoom digital camera (Olympus).

### RNA extraction and qRT-PCR

Total RNA was isolated in 3−5 dag seedlings with TriReagent (Life Technologies). Residual DNA contaminants were removed with RNase-free DNase (Roche). One microgram of RNA was used as template to synthesize the first cDNA strand with the iScript cDNA Synthesis Kit (Bio-Rad). Expression levels were analysed by qRT-PCR. Reactions were performed in 384 well-plates with a LightCycler real-time thermocycler (Roche).

### Statistical analysis

Statistical differences were assessed with student *t*-tests. All experiments were repeated two to three times with similar results. Error bars show standard errors of the mean (±SEM). Statistical significance are indicated as: *, *P*<0.05; **, *P*<0.01; ***, *P*<0.001.

## Results

### *GLV6* transcription is induced in LRFCs prior to their first division

Based on the characterization of promoter-reporter lines, we have previously shown that *GLV6* is transcribed in the RAM and is the first *GLV* transcript detected during primordium formation (Fernandez *et al.*, 2013*a*). The observation that *GLV6* transcription is already detected in stage I LRPs suggests that the encoded signalling peptide is involved in the very first steps of LR initiation.

**Fig. 1.**
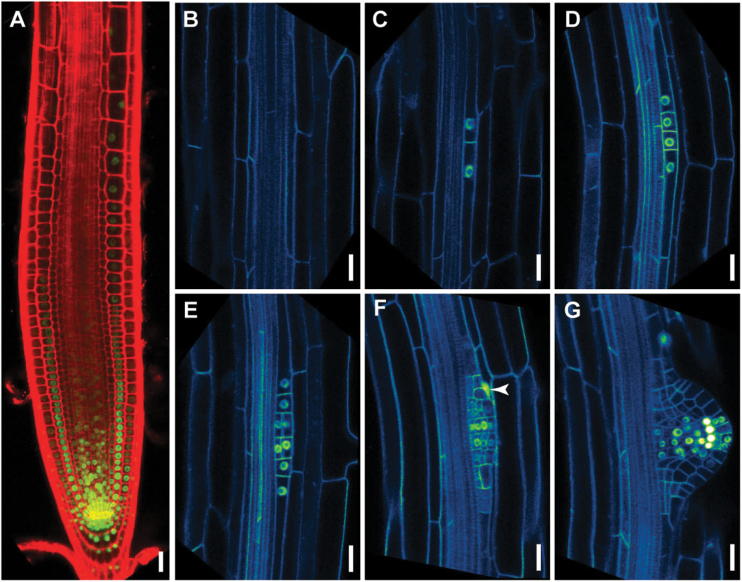
*GLV6* expression pattern during lateral root initiation. *GLV6* transcription was detected in a *GLV6pro:NLS-GFP-GUS* line. (A) *GLV6* transcription in the primary root. Cell walls were counterstained with propidium iodide (red). (B−G) *GLV6* expression at the site of lateral root initiation in plants carrying the *GLV6pro:NLS-GFP-GUS* marker together with the Wave 131Y plasma membrane reporter. (B) The *GLV6* signal is not detected in pericycle cells at the beginning of the differentiation zone. (C) The *GLV6pro*-driven GFP nuclear signal appears in founder cells prior to the first anticlinal division. (D−G) *GLV6* transcription remains active throughout primordium formation and is confined to the central cells at later stages. The arrowheads point to expression in the endodermal cell adjacent to the primordium. Scale bars, 20 µm.

To address this hypothesis, we first examined the timing of *GLV6* induction during primordium initiation in plants carrying the *GLV6pro::NLS-GFP-GUS* transgene, where a nuclear GFP signal indicates *GLV6* transcription (Fig. 1). To visualize cell boundaries, we introduced in these plants a plasma membrane marker (Wave 131Y; Geldner *et al.*, 2009) (Fig. 1B−G). Analysis of the reporter line in the maturation zone revealed that *GLV6* is only transcribed in pericycle cells where a primordium is forming and appeared also at later stages in the endodermal cell(s) surrounding the primordium. The GFP nuclear signal was first detected in paired pericycle cells with nuclei positioned close to the common cell wall suggesting that these are LRFCs (Fig. 1C), then after the first division in all primordium stages (Fig. 1D−G). At later stages, *GLV6* transcription was mainly restricted to the centre of the developing primordium (Fig. 1F, G).

We conclude from this data that *GLV6* transcription starts with the nuclear migration that marks LRP initiation, before the first asymmetric division, then persists throughout LRP development and extends into the overlying endodermal cells.

### *GLV6* mutants show defective lateral root formation

**Fig. 2.**
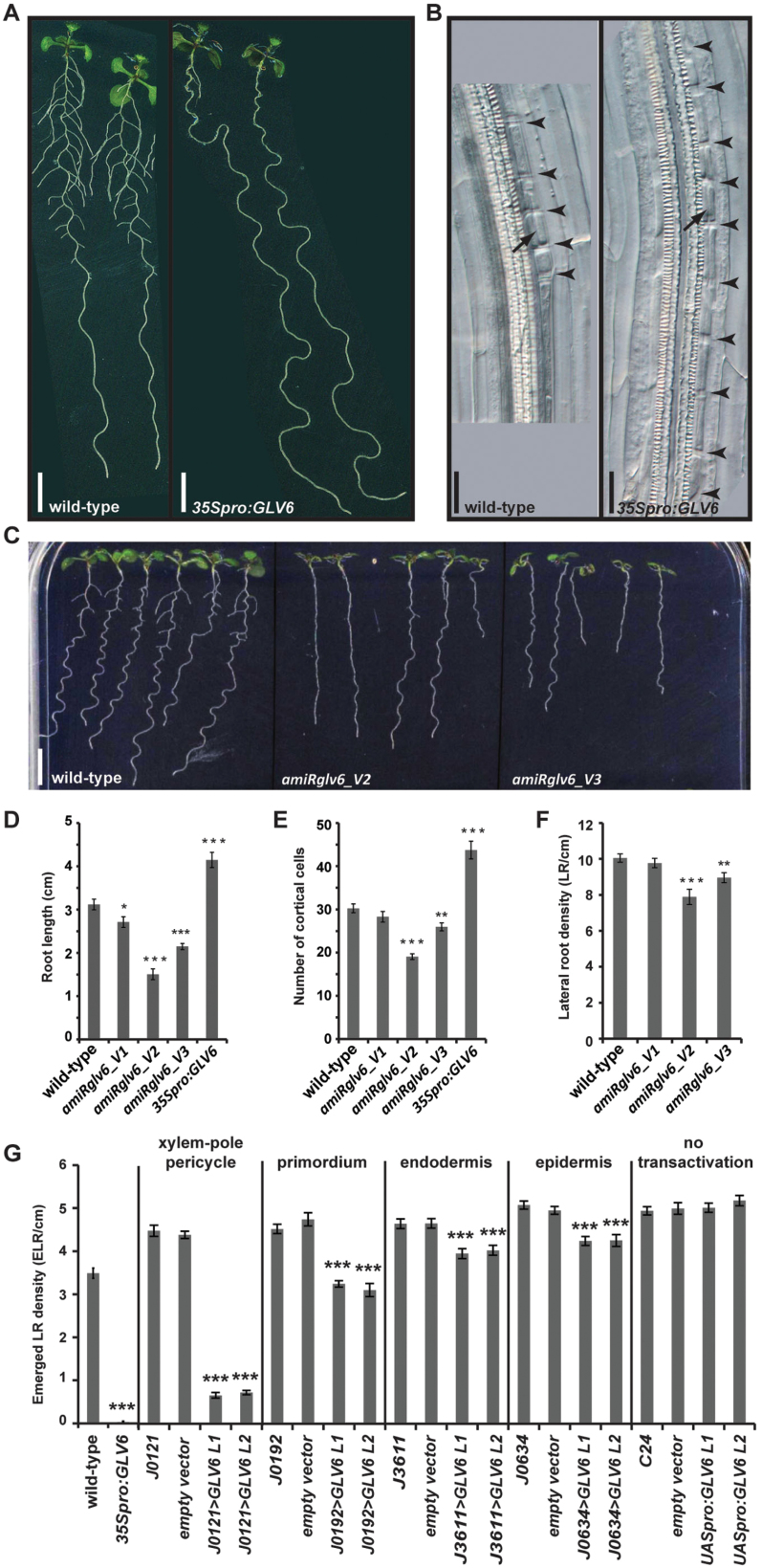
Lateral root phenotypes in *GLV6* mutants. (A) *GLV6* constitutive overexpression results in high reduction of ELR. (B) *GLV6* overexpression induces supernumerary pericycle cell divisions. Arrowheads and arrows point to anticlinal and periclinal divisions, respectively. (C) Phenotype of silenced *GLV6* lines. Quantification of root length (D) and RAM size (E) in *GLV6* silencing and overexpression lines. RAM size was determined as the number of cortical cells from the QC up to the first elongating cell (*n*=15−20). (F) Lateral root density in 9 dag seedlings (*n*=15). (G) Lateral root phenotype resulting from ectopic *GLV6* expression in different root cell layers. ELR density was measured in 12 dag T3 single-locus homozygous plants where *GLV6* overexpression is transactivated in different root cell layers (*n*=20). The *UASpro:GLV6* construct or an empty vector control was transformed into different enhancer trap lines (Supplementary Fig. 1) or the C24 wild type (no transactivation). Four to seven independent *GLV6* transactivation lines were obtained with similar phenotypes for each driver locus. For clarity, the data for only two lines are shown per driver (named L1 and L2). The asterisks indicate that the ELR density was significantly different (*P*<0.001) compared to the empty vector control. Scale bars: 0.5cm (A and C); 20 µm (B).

Next, we analysed the phenotype of plants that ectopically express the *GLV6* gene under the control of the constitutive *35S CaMV* promoter (*35Spro:GLV6*). We previously reported, and confirmed herein, that the *35Spro:GLV6* primary roots produced considerably fewer ELRs (Fernandez *et al.*, 2013*a*) (Fig. 2A). However, the lack of visible laterals was not caused by the absence of cellular activity. Indeed, the detailed microscopic analysis of *35Spro:GLV6* plantlets revealed that pericycle cells underwent several rounds of division at multiple sites along the differentiated primary roots (Fig. 2B). XPP cell files were characterized by excessive anticlinal divisions resulting in numerous cells along outstretched root segments. The size of these segments suggests that the borders between separate initiation sites could no longer be distinguished because the portions of the pericycle undergoing anticlinal divisions had probably expanded along the root axis and eventually merged. In addition it appeared that one single round of periclinal division produced a bilayered pericycle, but additional periclinal events were only rarely observed. Consequently, the formation of the stereotypical dome-shaped primordium did not occur, thereby preventing almost completely the normal formation of LRs and their emergence out of the primary root body.

To further study the role of *GLV6* in lateral root development, we searched for mutant lines in publicly available mutant collections. Unfortunately, no T-DNA line was found with an insertion in either the coding sequence or the promoter of the *GLV6* gene. Furthermore, the tilling of an EMS-mutagenized population (Till *et al.*, 2003; http://tilling.fhcrc.org/) did not identify alleles encoding premature stop codons or altering conserved protein sequences (data not shown).

We then silenced *GLV6* expression using artificial microRNA (amiRNA) (Schwab *et al.*, 2006). Three independent *amiRNAglv6* constructs were designed and assembled under the control of the endogenous *GLV6* promoter (V1−3, see ‘Materials and methods’ for details). Analysis of single locus homozygous *GLV6pro:amiRglv6* lines revealed that their primary root was shorter (Fig. 2C, D). In agreement with *GLV6* silencing levels (Supplementary Table S2), the loss-of-function phenotype was stronger for V2 and V3 than for V1 (Fig. 2D). The reduced root growth of *amiRglv6* lines might be indicative of GLV6 function in primary root growth and is consistent with the observed *GLV6* transcription pattern in QC cells and surrounding initials of the RAM (Fernandez *et al.*, 2013*a*) (Fig. 1A). Accordingly, RAM size as determined by the number of cortical cells, was significantly reduced in *amiRglv6* lines compared to wild-type. In contrast, *GLV6* overexpression resulted in increased root length and RAM size (Fig. 2D, E).

Considering all LRs, non-emerged and emerged, the total LR density was significantly reduced in *GLV6*-silenced lines V2 and V3 (Fig. 2F). Apart from that, we did not observe other defects in primordia at specific stages or in primordium shape (data not shown). That may be explained by the fact that other *GLV* genes are expressed at later stages (from stage II on) and could act redundantly with *GLV6* (Fernandez *et al.*, 2013*a*).

### Ectopic expression restricted to XPP cells is sufficient to trigger the *GLV6* LR *gof* phenotype

The specific expression of *GLV6* and the related LR overexpression phenotypes point to a function in early stages of primordium formation. However, because *GLV6* is ectopically expressed in all the root tissues of *35Spro:GLV6* lines, it is difficult to conclude whether the LR *gof* phenotype is a direct or a secondary effect. To confirm that the observed phenotype is caused by *GLV6* being ectopically transcribed in pericycle cells, we expressed the gene in different cell layers. For this purpose, the *GLV6* ORF was fused to an upstream activation sequence (*UAS*) and the resulting *UASpro:GLV6* construct was transformed in different GAL4-GFP enhancer trap lines where the GAL4 yeast transcription factor is expressed in specific tissues. The transactivation lines were selected on the basis of the GAL4-responsive green fluorescent protein (GFP) marker (Supplementary Fig. S1). Gene transcription is driven by J0121 in all XPP cells, from the elongation zone upward (Laplaze *et al.*, 2005; Parizot *et al.*, 2008); by J0192, only in the developing primordium, from stage I on (Laplaze *et al.*, 2005, 2007); by J3611, in the endodermis and at a lower level in the cortex in the more mature part of the root; and by J0634, in the epidermis starting at the beginning of the differentiation zone. All lines were chosen so that no transactivation was detected in the RAM to avoid secondary effects potentially caused by expressing *GLV6* in that region (Supplementary Fig. S1).

Several independent *UASpro:GLV6* homozygous lines were generated for each enhancer trap line and the number of ELRs was counted as an easily quantifiable phenotype characteristic of *GLV6 gof* (Fig. 2G and data not shown). Transactivation within all XPP cells (*J0121>GLV6*) yielded a severe phenotype similar to the one previously observed in *35Spro:GLV6* roots. In *J0192>GLV6* roots, the number of emerged LRs was also notably reduced although to a lesser extent than in *J0121>GLV6* seedlings. *J3611>GLV6* and *J0634>GLV6* roots displayed a milder phenotype.

Microscopic analysis revealed massive anticlinal divisions in the *J0121>GLV6* pericycle as in *35Spro:GLV6* roots. Excessive divisions were also observed in the other transactivation lines but the frequency and the length of root segments undergoing continuous anticlinal divisions decreased as *GLV6* overexpression was transactivated farther away from the pericycle (Supplementary Fig S2A). This is in agreement with the observed differences in emerged lateral root number since the probability to form dome-shaped primordia that develop into mature LRs is higher when anticlinal divisions are more restricted, similar to the wild-type.

Massive anticlinal divisions prevented the distinction of neighboring initiation events that cannot thus, be easily counted, especially in *J0121>GLV6* and *J0192>GLV6* lines. Therefore, instead of counting initiation sites with continuous divisions we quantified those where anticlinal divisions were restricted, reasoning that these were more likely to form a functional primordium and to reflect the number of emerged LRs. Thus, fewer primordia (or initiation sites with restricted divisions) correspond to longer and more frequent regions of excessive pericycle divisions and vice versa (Supplementary Fig. S2B). As expected, only a few primordia were observed in *J0121>GLV6* roots in agreement with the counted emerged LRs. *J0192>GLV6* and *J3611>GLV6* roots contained an intermediate number of restricted division sites, however these were usually longer in *J0192>GLV6* seedlings and therefore less likely to develop into mature LRs. Excessive pericycle divisions were also observed in some *J0634>GLV6* roots, but rarely compared to the other transactivation lines.

In conclusion, the phenotype was much less severe when the peptide was secreted from the epidermis, or the endodermis and cortex than from XPP cells. This observation agrees with the assumption that secreted peptides carry a molecular signal over short distances and confirms our hypothesis that the *GLV6* LR *gof* phenotype is mostly due to ectopic expression in XPP cells. The phenotypic difference observed between J0121 and J0192 transactivation can be explained by the fact that ectopic expression is gradually turned on after LRP initiation in J0192, while it is constitutively on in XPP cells of J0121 (Supplementary Fig. S1). The ranking of the transactivation phenotypes appears to reflect a distance effect rather than differences between driver promoter activity because the phenotype strength was similar among all independent transgenic lines analysed for the same enhancer trap locus, regardless of *GLV6* transcript level (Fig. 2G; Supplementary Fig S2B; Supplementary Table S2; data not shown). Consequently, we postulate that the GLV6 peptide is an autocrine signal because our data indicates that it is perceived by the LRFCs that produce it.

### The GLV6 conserved carboxyl-terminal domain carries the bioactive secreted signal

**Fig. 3.**
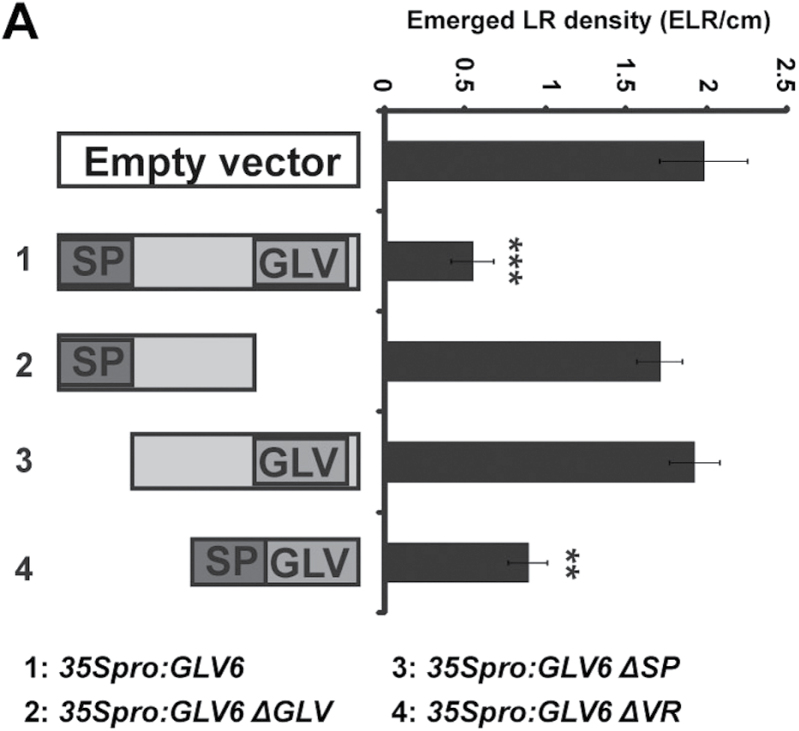
The GLV6 mature peptide is contained in the C-terminus of the precursor. Figure shows the ELR density in lines overexpressing truncated *GLV6* ORFs (*n*=12−28). SP, signal peptide; GLV, conserved C-terminal domain; VR, variable region. The asterisks indicate that the ELR density was significantly different compared to the empty vector control (**, *P*<0.01; ***, *P*<0.001).

Signalling peptides are translated as large precursors that are processed to yield the mature secreted peptides (Matsubayashi, 2011). Three domains can be distinguished in GLV precursor proteins, from the amino- to the carboxyl-terminus: a signal peptide (SP) presumably necessary for secretion, a variable region (VR), and a conserved GLV motif that defines the family (Fernandez *et al.*, 2013*b*). To confirm that the bioactive GLV6 signal peptide is encoded in the GLV motif, we overexpressed truncated versions of the *GLV6* ORF in *Arabidopsis* plants. The tested constructs included the *GLV6* sequences either (i) coding for the full precursor where the three aforementioned domains are present, (ii) lacking the region corresponding to the SP (*35Spro:GLV6* ***Δ*** *SP*), (iii) lacking the region corresponding to the GLV motif (*35Spro:GLV6* ***Δ*** *GLV*), or (iv) comprising a translational fusion between the SP and the GLV domains and thus where the variable region has been deleted (*35Spro:GLV6* ***Δ*** *VR*) (Fig. 3).

Roots overexpressing the truncated *GLV6* ORFs that lacked either the SP or GLV motif sequences produced the same number of ELRs as the control plants transformed with an empty vector (Fig. 3). However, overexpression of the *GLV6* sequences corresponding to a fusion between the SP and GLV domains, without the VR, was sufficient to significantly reduce the number of ELRs. These experiments confirm that both the SP and the GLV motif are necessary and sufficient for GLV6 function during LR formation and that the mature GLV6 peptide contained in the carboxyl-terminal region of the precursor is secreted from pericycle cells to activate a signalling pathway.

### GLV6 peptide application phenocopies *GLV6 gof* mutants

The endogenous GLV mature peptides have been identified for GLV1, GLV2, GLV3 and GLV11 (Matsuzaki *et al.*, 2010; Whitford *et al.*, 2012). In all four cases, the mature peptides have been found to be post-translationaly modified with sulfonation of a tyrosine and hydroxylation of a proline. In previous studies, we showed that tyrosine sulfonation is important for peptide bioactivity (Whitford *et al.*, 2012; Fernandez *et al.*, 2013*a*).

**Fig. 4.**
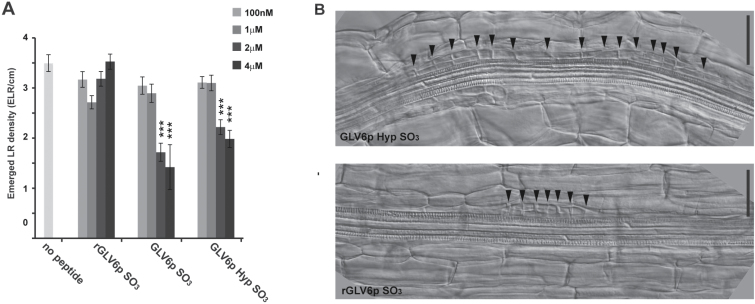
Treatments with GLV6 peptides phenocopy overexpression of the gene. (A) ELR density of seedlings treated with GLV6 synthetic peptides. The asterisks indicate statistically significant differences compared to the randomized rGLV6p SO_3_ control at the same concentration (*,*P*<0.05; **, *P*<0.01; ***, *P*<0.001) (*n*=25). (B) Treatment with GLV6 bioactive peptides induce pericycle divisions similar to those observed in *GLV6* overexpression lines. Peptides were added at 1 µM. Arrowheads indicate anticlinal divisions. Scale bars, 50 µm.

We then investigated whether LR development can be perturbed upon treatment with a synthetic sulfonated GLV6 peptide (GLV6p SO_3_). A random peptide (rGLV6p SO_3_) containing the same amino acids but in a randomized sequence was used as control. Seedlings germinated in liquid media supplemented with GLV6p SO_3_ had decreased ELR density compared to untreated plants or rGLV6p SO_3_ controls reminiscent of the phenotype observed in *GLV6* overexpression plants (Fig. 4A). Nevertheless, the reduction in emerged LR density resulting from peptide treatment, even at the highest tested concentration (4 µM), was not as strong as that of *GLV6* ectopic expression (compare Fig. 2G and 4A). Since the functional role of proline hydroxylation has not been reported so far for GLV peptides, we then tested whether it could further increase peptide bioactivity. Treatments with GLV6p Hyp SO_3_ had the same effect as those with GLV6p SO_3_, indicating that, at least in exogenous synthetic GLV6 peptides, the presence of a hydroxproline residue does not enhance bioactivity (Fig. 4A). As in *35Spro:GLV6* plants, excessive anticlinal divisions were observed in early stage LRPs of roots germinated in the presence of GLV6 SO_3_ or GLV6p Hyp SO_3_ peptides (Fig. 4B and data not shown).

### Excess GLV6 activity decreases the asymmetry of the initial founder cell division

**Fig. 5.**
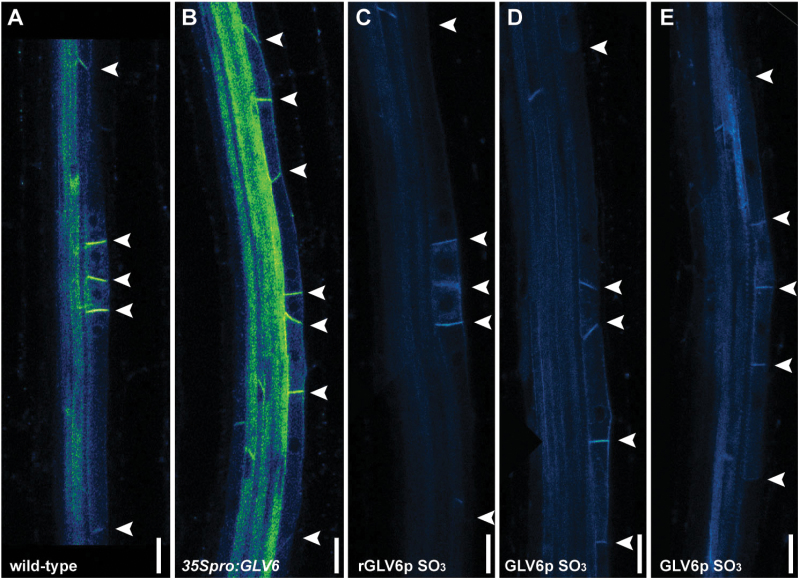
Excess GLV6 activity disrupts the first pericycle asymmetric divisions during primordium initiation. (A−E) The GFP signal in *PIN1pro:PIN1-GFP* plants was used as a plasma membrane marker. These plants were either crossed to *GLV6* overexpression plants (A, B) and the F1 progeny was analysed or they were germinated on 1 µM of the indicated peptide (C−E). Arrowheads indicate anticlinal divisions. Scale bars, 20 µm.

To further study GLV6 function in primordium initiation we used a *PIN1pro:PIN1-GFP* line as a plasma membrane marker to visualize the first divisions occurring in LRFCs since this auxin transporter is known to localize to the plasma membrane in the forming primordium (Benkova *et al.*, 2003). In untreated *PIN1pro:PIN1-GFP* plants and in plants treated with a random control peptide, the first asymmetric anticlinal division was clearly visible, resulting in two small central cells and two larger flanking cells as previously reported (Fig. 5A, C). However, a remarkably different pattern was observed when these plants were either crossed to *35Spro:GLV6* (Fig. 5B) or germinated on GLV6p SO_3_ (Fig. 5D, E), whereby the initial anticlinal divisions yielded daughter cells of more similar sizes. Sometimes, one of the two neighbouring LRFCs divided symmetrically while the other one divided asymmetrically (Fig. 5D).

**Fig. 6.**
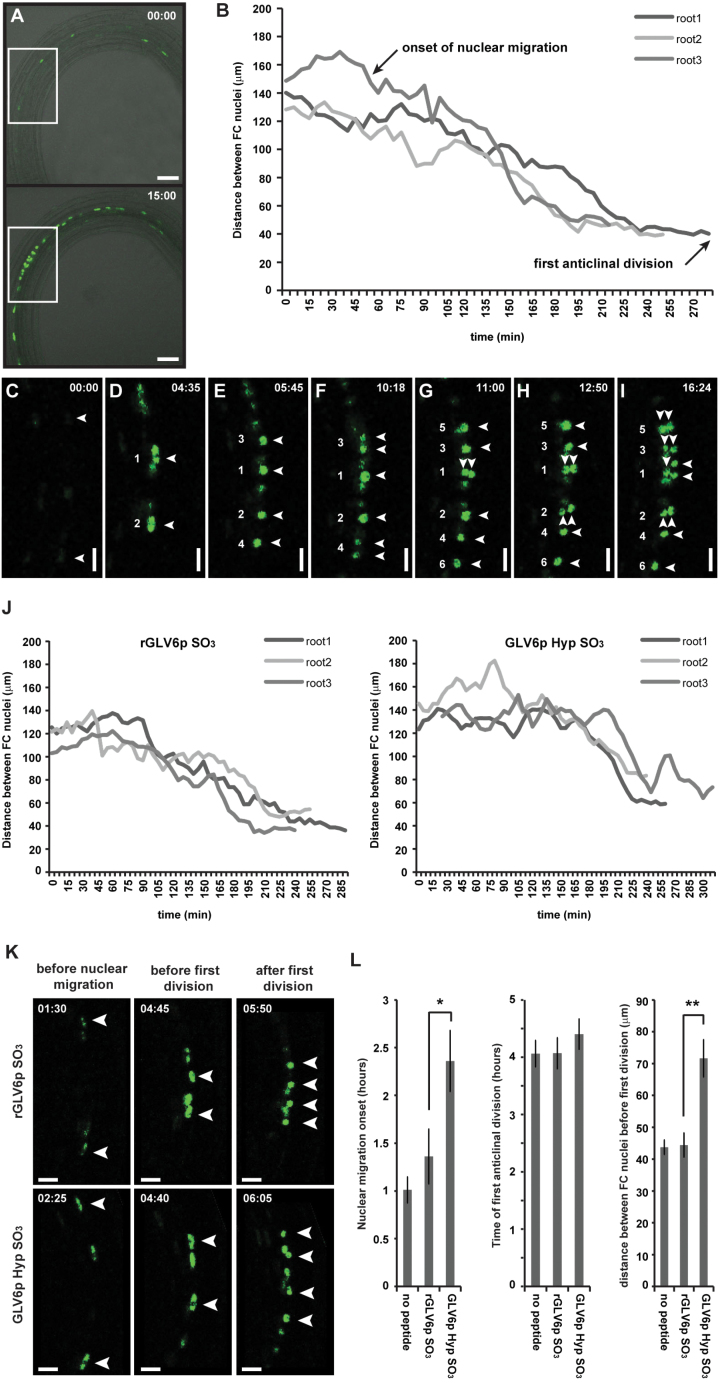
GLV6 peptide treatment affects nuclear migration before the first division of the founder cells. (A) Stage II primordium is observed 15h after mechanical bending of the *GATA23pro:NLS-GFP-GUS* primary root. (B) Dynamics of LRFC nuclei represented as the distance between the two nuclei over time, from the beginning of the experiment until the first anticlinal division. Three independent roots are shown. Arrows show timing examples of the indicated events. Higher magnification of the area framed in A shows different events taking place in LRFCs during primordium initiation: (C) immediately after bending; (D) after nuclear migration; (E) first anticlinal division; (F) second anticlinal division; (G, H) first periclinal division in central nuclei; (I) second periclinal division in flanking nuclei. Notice plane change again in central nuclei. (J) Effect of peptide treatment on the onset of nuclear migration. The distance between LRFC nuclei was measured from t_0_ up to the first anticlinal division in the presence of 1 µM of the indicated peptides. Three independent roots are shown. (K) Representative images showing beginning and end of nuclear migration and first anticlinal division in LRFCs treated with 1 µM of the indicated peptides. (L) Quantification of events illustrated in K. Charts show average time/distance ±SEM (*n*=6 to 8). Time is shown as h:min. All indicated time points refer to time after the first image was taken. Nuclei were followed in six to eight movies for each condition. Scale bars: 50 µm (A); 20 µm (C−I and K).

To determine how early GLV6 may function, it is necessary to detect the very first cellular events at the onset of LRP initiation. For this purpose, we used the *GATA23* gene whose transcription is induced when founder cells are specified in the elongation zone. Therefore, in a *GATA23pro:NLS-GFP-GUS* maker line, the founder cell nuclei can already be identified before the first cellular signs of LPR initiation as marked with a GFP signal (De Rybel *et al.*, 2010). As reported before, primordium formation can be induced by mechanical bending of the primary root (Ditengou *et al.*, 2008; Laskowski *et al.*, 2008). Thus, we manually bent the main root of *GATA23pro:NLS-GFP-GUS* seedlings and performed confocal time-lapse series for 14−16h, long enough for the appearance of a stage II primodium in the bent region (Fig. 6A).

In agreement with previous reports, we observed that during primordium initiation the nuclei of two adjacent pericycle cells migrate towards the common cell wall, then anticlinal and periclinal divisions give rise to the first stages of primordium development (Fig. 6C−I; Supplementary Movie S1). Thus, using this system, the LR initiation process can be analysed with high resolution up to stage II, including in response to peptide addition.

We then analysed the dynamics of primordium initiation without any treatment. First, we followed nuclear migration towards the common cell wall by measuring the distance between the two founder cell nuclei over time. The dynamics of nuclear migration after bending was strikingly similar between independent roots (Fig. 6B; Table 1). Parameters such as onset and end of nuclear rapprochement, as well as the final distance between the nuclei before the first anticlinal division, were highly reproducible (Fig. 6B, 6L; Table 1). Likewise, subsequent cell divisions took place at regular time intervals (Table 1). The time between the first and second anticlinal divisions was on average 4.7h (284min) (Table 1). Then, the division plane changed in central cells and the first periclinal division took place only 25min after (Fig. 6F−H; Table 1). We also remarked that the paired LRFCs are usually not exactly synchronized, and can divide a few minutes apart. In fact, this time lag is maintained in later divisions occurring in daughter cells regardless of the division plane, i.e. daughter cells of the earlier dividing founder cell keep dividing before descendants of the later dividing founder cell (Table 1). Our analysis of the initial LRP cell divisions suggests that this process is highly controlled and that it operates as a single functional module once launched.

**Table 1.** Timing of cellular events following lateral root primordium initiation

**Cellular events** ^a^	**No peptide**	**rGLV6p SO**_**3**_	**GLV6p Hyp SO**_**3**_
Nuclei initiate migration	61±8	82±18	142±19
Nuclei stop migration	199±20	206±20	233±13
First anticlinal division			
Cell position 1	244±14	244±16	264±16
Cell position 2	268±15	272±15	292±22
Second anticlinal division			
Cell position 3	527±24	510±19	541±20
Cell position 4	581±21	567±26	596±22
First periclinal division			
Cell position 1	552±28	551±14	579±25
Cell position 2	606±28	614±28	634±34

^a^ LRP initiation was triggered by root bending. Events were tracked in time-lapse image series. The position of the nuclei or cells refers to Fig. 6D, end of nuclear migration; Fig. 6E first anticlinal division; Fig. 6F, second anticlinal division; Fig. 6G and H, first periclinal division. The first dividing founder cell was given position 1. Cells with odd position numbers are descendants of the first dividing founder cell, those with even numbers of the second. No peptide (*n*=8); rGLV6p SO_3_, random sulfated GLV6 peptide (*n*=6); GLV6p Hyp SO_3_, GLV6 peptide containing a hydroxyproline and a sulfated tyrosine (*n*=6). Peptides were added at 1µm. Time after the first image is indicated in minutes ±SEM.

We then studied the effect of GLV6 peptide addition on the different cellular events. Seedlings were germinated on MS medium and peptides were applied to the root immediately after primary root bending (see ‘Materials and methods’). In untreated roots, the nuclei of founder cells started migrating towards each other ~1h after primary root bending and this polar movement lasted 2.3h (138min) on average (Table 1). Subsequently, nuclei stopped approaching and divided shortly after. In contrast, when LR initiation was induced in the presence of GLV6p Hyp SO_3_, the onset of nuclear migration was delayed by 1h compared to the rGLV6p SO_3_ control (Fig. 6J−L; Table 1; Supplementary Movie S2). Nevertheless, despite the late nuclear migration, founder cells in GLV6p Hyp SO_3_-treated roots divided approximately at the same time as in control experiments (Fig. 6K, L; Table 1). This data indicate that, at least in stages I and II, GLV6 excess does not alter the progression of the cell cycle but rather disrupts the division pattern. In addition, the distance between founder cell nuclei before the first division was larger when roots were treated with GLV6-derived peptides which is another proof of the loss of asymmetry caused by GLV6 activity (Fig. 6K, L). This result is in agreement with the defects observed in *gof* mutants where the wild-type division pattern is disturbed resulting in more symmetric divisions.

## Discussion

LRs are formed through a process that comprises several steps, the first one known to date occurring in the basal meristem. As XPP cells differentiate, some of them undergo a series of developmental programmes to eventually give rise to a mature lateral root. Despite numerous studies on LR development, many aspects of each of these steps remain unknown. In this report, we showed that GLV6 activity could be an important factor involved in primordium initiation. *GLV6* expression starts in LRFCs during the nuclear migration indicating that the GLV6 signal is produced very early, that is, before the first anticlinal division takes place. Furthermore GLV6 excess disturbs the initial asymmetric divisions.

The analysis of *amiRglv6* plants revealed that the root length is shorter in agreement with a reduced number of cortical cells in the RAM, while *GLV6* overexpression resulted in the opposite phenotype. This data, together with *GLV6* transcription in the QC and stem cells, indicate a function for *GLV6* in RAM homeostasis, possibly redundant with other *GLV* genes (Matsuzaki *et al.*, 2010).

Due to the inhibition of root growth in *amiRglv6* seedlings the analyses of LR formation is not straightforward. Nevertheless our results indicate a reduction in the total number of lateral roots. Our hypothesis is that a threshold level of GLV6 activity is necessary to trigger the first anticlinal asymmetric division and thus, lower GLV6 levels will result in less anticlinal divisions during LR initiation (reflected in reduced number of initiation events) while increased GLV6 levels would produce excessive anticlinal divisions and disturb the asymmetric pattern. The fact that short peptide treatments resulted in delayed nuclear migration immediately after LR induction indicates that the defects in asymmetric divisions observed in *GLV6* overexpression lines is not a secondary effect. However, it is important to notice that we have no evidence of GLV6 directly affecting the nuclear movement itself and therefore the differences observed in nuclear migration upon peptide treatment can only be considered as the read out of GLV6 action in the initial events of primordium formation. The time of the first divisions after LR induction was approximately the same between untreated and GLV6p-treated roots, confirming that GLV6 addition does not disturb the onset of cell division but rather interferes with the division pattern. Based on our data, it is tempting to speculate that GLV6 might be involved in cell polarity, for example by controlling a polarizing cue. However additional insights into its signalling pathway are needed to confirm this hypothesis.

The induction of *GLV6* transcription pattern coincides, spatially and temporally, with the auxin maximum that is first formed at the beginning of the LRP initiation and that is maintained at the core of the primordium throughout its development (Benkova *et al.*, 2003). The understanding of the GLV function in LR development will therefore require a careful analysis of the potential links between peptide and auxin signalling pathways, as such connections have already been described in other developmental programmes (Whitford *et al.*, 2012).

*GLV6* is transcribed in LRFCs and GLV6 *gof* lines show a defect in a process taking place in the same cells. Deleting the signal peptide from the precursor results in the loss of the overexpression phenotype indicating that the peptide must be secreted. In addition, *GLV6* phenotype is stronger when the gene is ectopically expressed in the pericycle compared to other cell layers. Therefore, we conclude that GLV6 is an autocrine signal. Furthermore, GLV6 could be part of a communication mechanism taking place between the two founder cells to coordinate their division pattern. Interestingly *GLV6* overexpression from the adjacent endodermal cells results in a much milder *gof* phenotype. This could be explained in several ways: (i) the Casparian strip may filter out (part of) the peptide before it reaches the pericycle cells; (ii) the peptide secreted from the endodermis may partially diffuse away as only one side of the endodermal cells is in direct proximity with the pericycle; (iii) a GLV6 peptide gradient resulting from its secretion by the neighbouring LRFCs controls a polarity cue and thus, the asymmetry of the initial divisions in the wild type. In this case, excess of peptide perceived at one cell side (secreted from endodermis), as opposed to all cell sides (secreted from XPP), will perturb cell polarization less.

Two post-translational modifications have been described in mature GLV peptides: tyrosine sulfonation and proline hydroxylation. It is known that sulfonation increases peptide bioactivity (Whitford *et al.*, 2012; Fernandez *et al.*, 2013*a*). In this report we showed that peptide bioactivity is not further increased by the presence of a hydroxylated proline in synthetic peptides. One possibility is that the hydroxyproline in GLV peptides is further modified with glycosylations as has been shown for other signalling peptides such as CLV3 and PSY (Amano *et al.*, 2007; Ohyama *et al.*, 2009). Nevertheless, despite repeated attempts to purify the native GLV6 peptide from media conditioned with *GLV6* overexpression plants (Ohyama *et al.*, 2008), we have been unable to detect any GLV6-related sequence (data not show). One reason for this failure could be that the high arginine content in the predicted mature GLV6 peptide sequence prevents peptide detection by mass spectrometry (Foettinger *et al.*, 2006).

As we studied the role of GLV6 in the early divisions of lateral root formation, we developed a new system to track primordium initiation with high time and space resolution. Combining root mechanical bending and the *GATA23pro:NLS-GFP-GUS* reporter line, we were able to follow and describe the dynamics of nuclear migration and the initial divisions after LR induction. Our results indicate that the first divisions leading to primordium initiation follow a regular pattern. In another study, Lucas *et al.* (2013) found that cell divisions during primordium formation are not stereotypical. Although we agree that after stage II, some cell divisions may become more randomly oriented, our exhaustive analysis of LRP initiation indicates that nuclear migration, the initial anticlinal asymmetric and the first periclinal divisions are stereotypical in the wild type, at least after induction by primary root bending. Furthermore using nuclear movement as a read out, we showed that GLV6 peptide treatment interferes with the first steps of primordium initiation. We foresee that this experimental setup will also be suitable to study the influence of other factors involved in early steps of primordium development.

To conclude, we have described new aspects of the dynamics of primordium initiation and presented evidence indicating that the GLV6 peptide is an important factor controlling this process. We hope our work will form the basis for future studies of LR development and can contribute to the understanding of the role of signalling peptides in the control of plant developmental processes.

## Supplementary data

Supplementary data are available at *JXB* online.

Supplementary Figure S1. Enhancer trap lines used for transactivation of *GLV6* expression in different root cell layers.

Supplementary Figure S2. Pericycle divisions caused by ectopic *GLV6* expression in different root cell layers.

Supplementary Movie S1. Early events during primordium formation visualized with the *GATA23:NLS-GFP-GUS* line.

Supplementary Movie S2. Effect of GLV6 peptide addition on lateral root initiation.

Supplementary Table S1. Primers used to generate truncated *GLV6* open reading frames.

Supplementary Table S2. *GLV6* RNA fold change in gain- and loss-of-function mutants.

Supplementary Data

## Abbreviations:

dag: days-after-germination
ELR: emerged lateral root
gof: gain-of-function
LRFC: lateral root founder cell
LRP: lateral root primordium
ORF: open reading frame
RAM: root apical meristem
SP: signal peptide
UAS: Upstream Activating Sequence
VR: variable region
XPP: xylem pole pericycle.
