# Supplementary material for: The GLV6/RGF8/CLEL2 peptide regulates early pericycle divisions during lateral root initiation
Source: J Exp Bot. 2015 Jul 10;66(17):5245–56. doi: 10.1093/jxb/erv329 (PMC4526922; doi:10.1093/jxb/erv329)
Supplement: Supplementary Data [file supp_66_17_5245__index.html]

The GLV6/RGF8/CLEL2 peptide regulates early pericycle divisions during lateral root initiation — Supplementary Data 

# The GLV6/RGF8/CLEL2 peptide regulates early pericycle divisions during lateral root initiation

## Supplementary Data

Data files

- Suppl\_Movie1.avi - Supplementary Data
- Suppl\_Movie2.avi - Supplementary Data
- jexbot148486\_file003.pdf - Supplementary Data
