# Supplementary material for: The GLV6/RGF8/CLEL2 peptide regulates early pericycle divisions during lateral root initiation
Source: J Exp Bot. 2015 Jul 10;66(17):5245–56. doi: 10.1093/jxb/erv329 (PMC4526922; doi:10.1093/jxb/erv329)
Supplement: Supplementary Data [file supp_erv329_jexbot148486_file003.pdf]

The GLV6/RGF8/CLEL2 peptide regulates early pericycle divisions during lateral root initiation

Ana Fernandez, Andrzej Drozdzecki, Kurt Hoogewijs, Valya Vassileva, Annemieke Madder, Thomas Beeckman, and Pierre Hilson

Supplemental Files

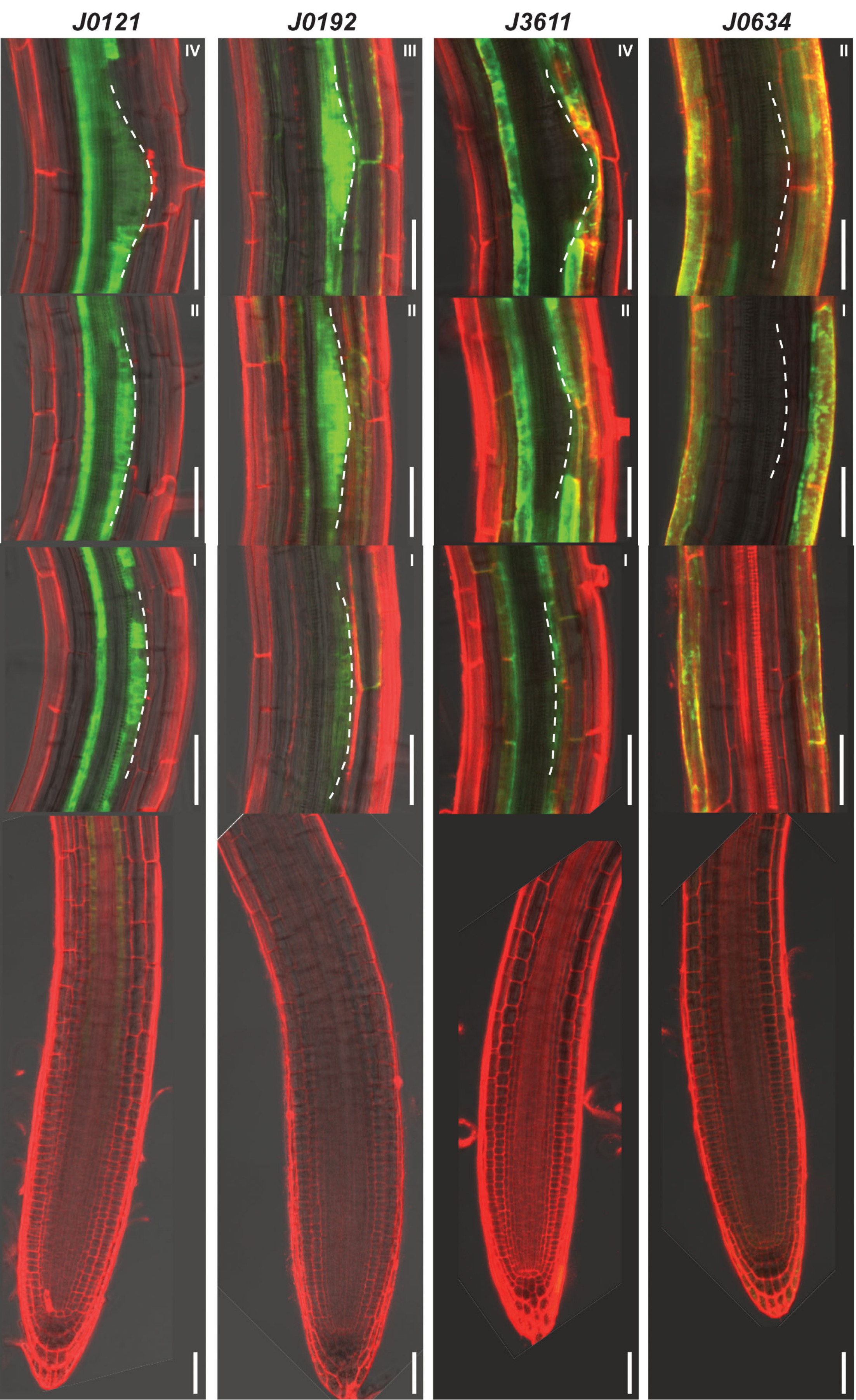
J0121
J0192
J3611
J0634
IV
III
IV
II
II
II
II
I
I
I
I

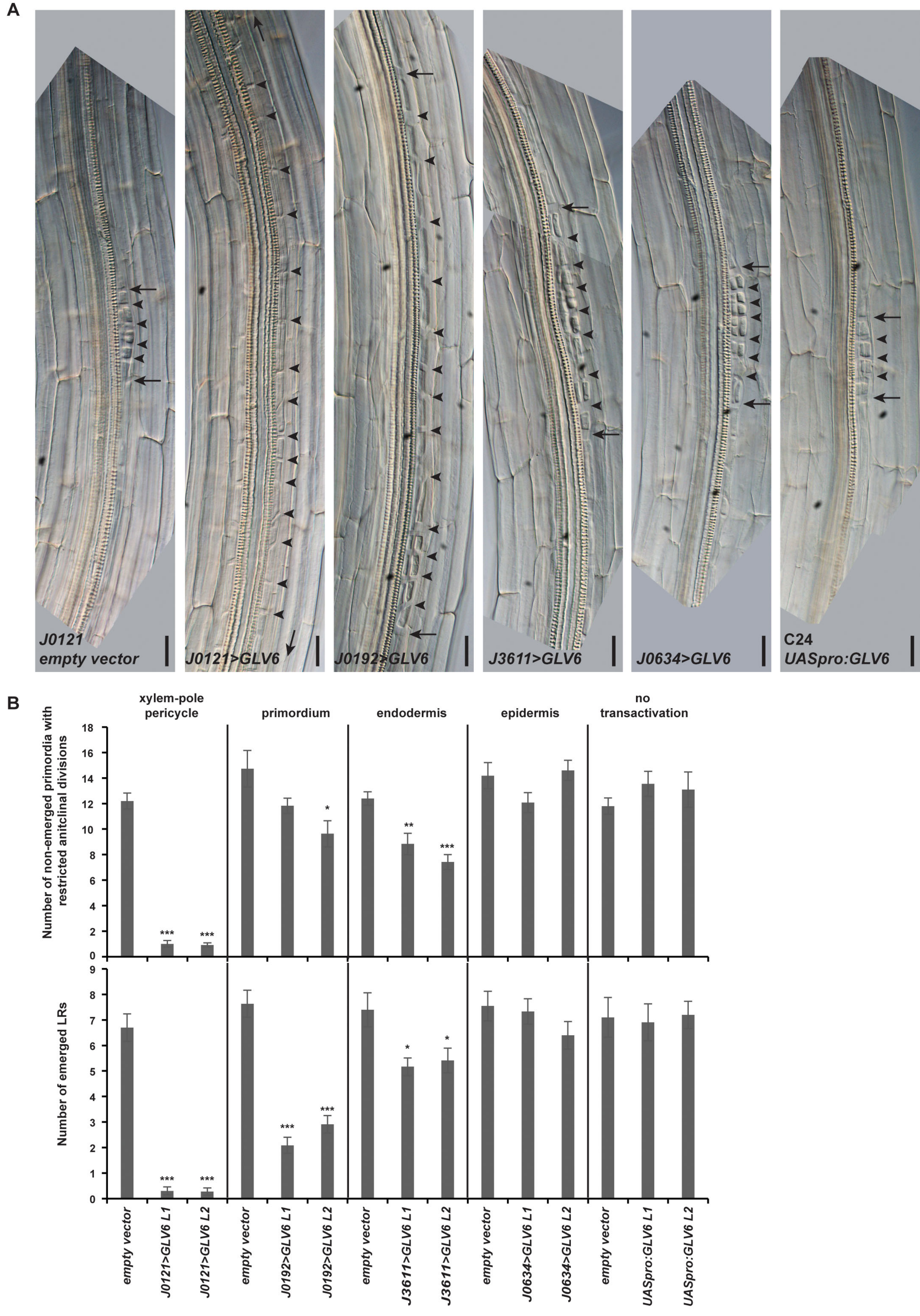
A
J0121
empty vector
J0121>GLV6
J0192>GLV6
J3611>GLV6
J0634>GLV6
C24
UASpro:GLV6
B
xylem-pole pericycle
primordium
endodermis
epidermis
no transactivation
Number of non-emerged primordia with restricted anitclinal divisions
Number of emerged LRs
empty vector
J0121>GLV6 L1
J0121>GLV6 L2
J0192>GLV6 L1
J0192>GLV6 L2
J3611>GLV6 L1
J3611>GLV6 L2
J0634>GLV6 L1
J0634>GLV6 L2
UASpro:GLV6 L1
UASpro:GLV6 L2

Table S1. RNA fold change in *GLV6* mutants. *GLV6* levels were measured by qRT-PCR in single locus homozygous lines. *GLV6* transactivation plants were compared to the same driver transformed with an empty vector. *AmiRglv6* plants were compared to the wildtype.

**Line**	***GLV6* RNA Fold change compared to control**
J0121 empty vector	1
J0121>GLV6_L1	44.59
J0121>GLV6_L2	38.14
J0192 empty vector	1
J0192>GLV6_L1	3.44
J0192>GLV6_L2	2.92
J3611 empty vector	1
J3611>GLV6_L1	31.28
J3611>GLV6_L2	58.47
J0634 empty vector	1
J0634>GLV6_L1	17.86
J0634>GLV6_L2	29.74
C24 empty vector	1
UASpro:GLV6_L1	1.82
UASpro:GLV6_L2	0.99
wildtype	1
amiRglv6_V1	0.88
amiRglv6_V2	0.47
amiRglv6_V3	0.29

Table S2. Primer sequences used to generate *GLV6* truncated open reading frames. The *GLV6ΔVR* construct was obtained through overlapping PCR as previously described (Atanassov *et al.*, 2009) by combining the *GLV6* primers listed below (*GLV6ΔVR* Fw and Rev) and the GWM13 primers, with pEN-L1-*GLV6*-L2 as template. Gateway *attB* sequences are underlined.

**Line**	**Primers sequence**
GLV6ΔGLV_Fw	GGGGACAAGTTTGTACAAAAAAGCAGGCTTATGAAGCTAATTAGAGTCACCC
GLV6ΔGLV_Rev	GGGGACCACTTTGTACAAGAAAGCTGGGTTTATTTCGCTAGAGCTTTTTTCTCTTC
GLV6ΔSP_Fw	GGGGACAAGTTTGTACAAAAAAGCAGGCTTATGCTACAACTTAAACATCCCTACTC
GLV6ΔSP_Rev	GGGGACCACTTTGTACAAGAAAGCTGGGTTTCATGGATCAAGAGGTAAAGC
GLV6ΔVR_Fw	CTTGTAACTCCAACATCTTCTCTAAAATATCTATCGATGGATTATCG
GLV6ΔVR_Rev	CGATAATCCATCGATAGATATTTTAGAGAAGATGTTGGAGTTACAAG
